# Streptococcus Viridans meningitis in an immunocompetent child: A case report

**DOI:** 10.1002/ccr3.7058

**Published:** 2023-03-08

**Authors:** Ali Goudarzi, Roham Sarmadian, Parsa Yousefichaijan, Abolfazl Gilani

**Affiliations:** ^1^ Iranian Center of Neurological Research Neuroscience Institute, Tehran University of Medical Sciences Tehran Iran; ^2^ Infectious Diseases Research Center Arak University of Medical Sciences Arak Iran; ^3^ Department of Pediatrics Arak University of Medical Sciences Arak Iran; ^4^ Department of Pediatric Surgery Tehran University of Medical Sciences Tehran Iran

**Keywords:** gram‐positive bacteria, meningitis, Viridans streptococci group

## Abstract

In bacterial meningitis, the Viridans streptococci group is not considered a prevalent pathogen. In contrast, the S. viridans group may cause endocarditis and fatal infections in immunocompromised children and adults. We report a 5‐year‐old immunocompetent boy with signs of meningitis. The CSF tested positive for meningitis with streptococcus viridans.

## Abstract 2

Streptococcus viridans meningitis can occur even in a healthy immunocompetent child without a clear history of predisposing factors like trauma or CNS surgery. Commonly reported bacterial meningitis pathogens should not lead us to completely rule out these uncommon causes, even in the absence of demonstrable evidence.

## 1 INTRODUCTION

Viridans streptococci are gram‐positive subgroups of α‐hemolytic streptococci that commonly reside in the oral cavity and the gastrointestinal tract. They are a common cause of dental caries and abscesses. Moreover, they could give rise to subacute bacterial endocarditis and severe infections in immunocompromised children and adults.^1^, ^2^ Viridans streptococci cause up to 3% of all cases of meningitis among adults. According to a study, they are responsible for 2% of meningitis cases in children with underlying medical conditions^3^, ^4^ Early diagnosis and treatment are the cornerstones of managing streptococcus viridans (S. viridans) meningitis in children. To our knowledge, there are few reports of S. viridans meningitis in immunocompetent children.^5^ Here, we describe an effectively treated 5‐year‐old healthy child with meningitis caused by S. viridans bacteria.

## 2 CASE PRESENTATION

The patient, a 5‐year‐old boy, presented to our hospital suffering from headaches and recurrent vomiting. He was the family's first baby born by natural vaginal delivery at 30 weeks of gestation (preterm). His birthweight was 1200 g, and he had no history of neonatal disease. He had eye strabismus correction surgery at the age of 3. His developmental milestones were normal up to now. He was admitted with symptoms like fever (38.5°C), recurrent vomiting, weakness, loss of appetite, and photophobia. The patient's parents did not report diarrhea or constipation. Before admission, he received fluid therapy and supportive treatment but the symptoms persisted. Upon arrival, he was awake and conscious, but weak; vital signs detected were as follows: 120 beats per minute, 30 breaths per minute, and blood oxygen saturation was 95%.

The physical examination revealed moderate nuchal rigidity and a positive Brudzinski sign. Cardiovascular and respiratory findings were unremarkable. A funduscopy and eye examination revealed no abnormal findings or papilledema. The differential diagnosis for the patient included meningitis and meningoencephalitis. A sterile lumbar puncture (LP) was conducted for him. Ceftriaxone and vancomycin regimen was started along with acyclovir and dexamethasone. Neutrophil‐dominant leukocytosis was observed along with normal erythrocyte sedimentation rate (ESR) and C‐reactive protein (CRP) values. The electrolyte results did not indicate any abnormalities. The results of a brain computed tomography **(**CT) scan were unremarkable. Based on the normal results of the brain magnetic resonance imaging (MRI), acyclovir and dexamethasone were discontinued. Clear cerebrospinal fluid (CSF) analysis showed 130 white blood cells with 96% neutrophils; glucose level was 49 mg/dL, and protein was 121 mg/dL. There was a rise in ESR to 53 mm/h in 3 days. CSF culture was positive for Streptococcus viridans, which was sensitive to vancomycin and resistant to clindamycin, erythromycin, and gentamycin. Both blood and urine cultures were negative. Echocardiography did not find any abnormalities. After 10 days of treatment, the symptoms subsided and he was discharged in stable condition.

At the patient's two‐week follow‐up appointment, the parents did not report any clinical complaints. Blood tests for the patient had reached normal levels.

## 3 DISCUSSION

Various causes such as bacterial infections or immune conditions can lead to meningitis, the inflammation of the membranes surrounding the central nervous system. Common organisms of bacterial meningitis differ based on age; E. coli and Group B streptococci are prevalent among neonates. In older children and adults, the prevalence of S. pneumonia, Neisseria meningitis, and Hemophilus influenza increases^6^, ^7^, ^8^ Viridans streptococci are rare causes of meningitis. Nevertheless, they are a common cause of infective endocarditis and critical infections in immunocompromised populations. A trauma or surgical procedure could result in an S. viridans infection in an adult.^8^, ^9^ Furthermore, poor oral hygiene might be a risk factor for S. viridans infections. Our patient had strabismus correction eye surgery; to date, there has been no history of meningitis due to S. viridans after an eye operation; however, endophthalmitis and meningitis due to S. pneumonia have been reported.^4^, ^10^

The presence of a single positive streptococcal meningitis culture from CSF or blood is considered contamination; however, in our case, we had clear symptoms as well as a positive extraction and successful treatment that pointed to the presence of S. viridans. In our patient, the glucose level was nearly normal, which is indicative of the fact that glucose levels in S. viridans meningitis can vary. One study found that half of the patients with S. viridans meningitis had a previous otolaryngeal infection as a possible transmission route, but this was not the case in our patient.^4^

ESR values in our patient increased gradually and were found to be normal at the time of admission. As an inflammation indication factor, we must consider that a normal ESR could not possibly rule out an active infection. In patients with bacterial meningitis, cerebral subdural empyema (SDE) is uncommon, although infections caused by S. viridans are more likely to induce the condition (which did not develop in this patient).^4^

Ceftriaxone plus vancomycin is the routine empiric meningitis therapy in our hospital setting. In this case, it was sensitive to vancomycin. Past antibiotics utilized in the treatment of S. viridans infection include penicillin G, aminoglycosides, ceftriaxone, and vancomycin.^4^, ^11^ but an ongoing resistance to antibiotics especially to penicillin has been reported in S. viridans meningitis.^12^ After ten days of treatment, we achieved successful results in treating our patient with ceftriaxone and vancomycin.

## 4 CONCLUSION

As a clinician dealing with a child presenting with symptoms of meningitis, we should not ignore streptococcus viridans as a possible pathogen even in a case with normal immune function and no clear history of trauma, cranial surgery, or otolaryngeal and paranasal sinus infection. Commonly documented bacterial meningitis pathogens should not lead us to exclude these unusual causes, even in the absence of their predisposing factors.

## AUTHOR CONTRIBUTIONS

**Ali Goudarzi:** Writing – original draft. **Roham Sarmadian:** Writing – original draft. **Parsa Yousefichaijan:** Supervision. **Abolfazl Gilani:** Investigation; project administration; resources; supervision; visualization; writing – original draft; writing – review and editing.

## FUNDING INFORMATION

There is no funding to the present study.

## CONFLICT OF INTEREST STATEMENT

The authors have no conflict of interests to declare.

## ETHICS STATEMENT

Our institution does not require ethical approval for reporting individual cases or case series. All of the authors declare that confidentiality of the patient was respected.

## CONSENT

Written informed consent was obtained from a legally authorized representative(s) for anonymized patient information to be published in this article.

## Data Availability

The data that support the findings of this study are available on request from the corresponding author.
